# Prognostic Impact of Platelet-Large Cell Ratio In Myelodysplastic Syndromes

**DOI:** 10.3389/fonc.2022.846044

**Published:** 2022-04-01

**Authors:** Qiuni Chen, Yue Chen, Yijing Zhang, Lijuan Zhang, Kankan Chen, Zhengmei He, Chunling Wang, Liang Yu

**Affiliations:** ^1^ Department of Hematology, The Affiliated Huaian No.1 People’s Hospital of Nanjing Medical University, Huaian, China; ^2^ Key Laboratory of Hematology of Nanjing Medical University, Nanjing, China

**Keywords:** myelodysplastic syndromes (MDS), platelet–large cell ratio (P-LCR), survival, prognosis, biomarker

## Abstract

**Background:**

Myelodysplastic syndromes (MDSs) are a very heterogeneous group of myeloid disorders with high prevalence and risk of developing acute myeloid leukemia. The more accurate risk stratification can provide a better guidance of treatment. The platelet–large cell ratio (P-LCR) is a parameter reported in complete blood cell count tests, and was associated with many diseases, but its role in MDS is not clear.

**Purpose:**

This study aims to explore the impact of the P-LCR on the prognosis of patients with MDS, which is of great significance for clinical treatment.

**Methods:**

In the retrospective study, 122 newly diagnosed MDS patients were enrolled. We used the bioinformatics tool X-tile to define a P-LCR threshold of 36.7% to predict prognosis. Patients were divided into P-LCR^low^ and P-LCR^high^ groups, and their characteristics were compared between the two groups.

**Results:**

Results show that the P-LCR^low^ was associated with worse overall survival (OS) than the P-LCR^high^ patients (median OS, 18.53 months versus 25.77 months, p=0.0057), but there were no statistical differences in progression-free survival (PFS) between the two groups (p=0.2001). The results of univariate and multivariate Cox proportional hazard analyses adjusted for gender, bone marrow blast level, platelet count, and International Prognostic Scoring System scores showed that the P-LCR was useful in the evaluation of PFS [hazard ratio (HR) 0.212, 95%CI 0.064–0.702, p=0.011] and OS of MDS (HR 0.464, 95%CI 0.284–0.757, p=0.002).

**Conclusion:**

This study is the first report showing that the P-LCR would be a simple and immediately available biomarker for predicting the prognosis of MDS.

## Introduction

Myelodysplastic syndromes (MDS) are one of the most prevalent hematological malignancies originating from hematopoietic stem/progenitor cells. It is a very heterogeneous group of myeloid disorders characterized by somatic mutations in hematopoietic stem cells, leading to ineffective hematopoiesis, bone marrow dysplasia, and an increased risk of transformation to acute leukemia (1).

The prevalence of MDS increases with age. The choice and timing of therapy depend on the risk stratification. The International Prognostic Scoring System (IPSS) (2) and revised International Prognostic Scoring System (IPSS-R) (3) are the most commonly used prognostic models. In general, all these scoring systems include the analyses of peripheral cytopenias, percentage of blasts in the bone marrow, and cytogenetic characteristics. Patients with MDS are generally divided into two different broad subgroups according to two scoring systems: lower- and higher-risk groups. Patients with a lower risk group by IPSS are those with low and intermediate-1(INT-1) diseases and very low, low, and some subsets of intermediate risk by the IPSS-R. Patients with a higher-risk disease are those with intermediate-2 (INT-2) and high risk by the IPSS and some subsets of intermediate-, high-, and very high-risk disease by the IPSS-R. Although the IPSS and IPSS-R are very important and serve as part of the main eligibility criteria for past and ongoing registration in clinical trials, many limitations remain.

With the deepening of biomarker research, various types of indicators, including genetics, blood parameters, and even nutritional indexes, had been found to be related to the biological characteristics and even the prognosis of MDS (4–6). Therefore, the concise indexes may help to more accurately evaluate the prognosis of MDS.

The platelet large–cell ratio (P-LCR) is an index representing the percentage of platelets larger than 12 fl in the circulating pool (7), generated by the automatic blood cell counter, thus probably identifying those platelets that are metabolically and enzymatically more active than small platelets, and it is easily available at an affordable cost. The original description of this parameter is from 1981 (7). It is determined based on flow cytometry and automated blood cell analysis techniques. The P-LCR was the best tool to assess megakaryocyte activity and a good monitoring tool for platelet activity. Evidence has indicated that the P-LCR contributes to the diagnosis of certain diseases (8–10). For instance, the P-LCR was proven higher in patients with chronic myeloid leukemia than patients with reactive thrombocytosis, essential thrombocythemia, or polycythemia vera (11). In clinical practice, abnormal platelet parameters are relatively common in MDS patients at initial diagnosis, but its impact on the prognosis remains unclear.

In this study, we performed a retrospective review to evaluate whether the P-LCR is associated with the prognosis of MDS.

## Materials and Methods

### Study Design and Patient Selection

One hundred and sixty-four patients with newly diagnosed MDS between March 2010 and January 2021 were reviewed in the Huai’an No.1 People’s Hospital. MDS was defined according to the World Health Organization (WHO) 2008 and 2016 classification for MDS. The study population was selected according to the following criteria and followed up to April 2021.The study was approved by the Institutional Review Committee of Huai’an No.1 People’s Hospital and implemented in conformity with the Declaration of Helsinki. All the patients were anonymous. Informed consent was waived because of the retrospective design of the data collection.

The inclusion criteria are as follows: a) diagnosed with MDS according to the 2008 and 2016 WHO definitions; b) complete blood samples were obtained at diagnosis and before any interventions; and c) detailed clinical data were available.

The exclusion criteria are as follows: a) age < 18 years; b) platelet transfusion before the measurement of the P-LCR; and c) active bleeding symptoms.

### Measurement of Platelet–Large Cell Ratio

Whole-blood samples collected into tubes were used for the measurement of the P-LCR. The baseline P-LCR level at diagnosis was defined as the value that was obtained on the nearest day before the diagnosis. The P-LCR was measured using XN-9000 (Sysmex, Kobe, Japan). The reference range for the P-LCR in our institution is 13.0%–43.0%. We used the bioinformatics tool X-tile to define a P-LCR threshold of 36.7% to predict prognosis (12). Subjects were classified as P-LCR low (<36.7%; N = 74) or P-LCR high (≥36.7%; N = 48) cohorts.

### Statistical Analyses

Data analyses were performed with the Statistical Package (SPSS 26.0 Inc., Chicago, IL, United States) and Graphpad Prism 6 (Graphpad Software, California, United States). The differences of categorical variables between groups were made by using the Mann–Whitney U-test or chi-squared test. Kaplan–Meier analysis was used to assess the associations of the P-LCR with progression-free survival (PFS) and overall survival (OS). PFS, the primary end point, was defined as the duration from the first treatment to the progression of MDS, death of any cause, or end of clinical follow-up. OS, the secondary end point, was defined as the duration from the first treatment to all-cause death or the end of follow-up. Kaplan–Meier survival curves were performed to compare the prognosis between the P-LCR^low^ and P-LCR^high^ groups by the log rank test. The X-tile software (Version 3.6.1; Yale University, New Haven, CT, United States) was conducted to evaluate the optimal cut-off P-LCR. Univariate and multivariate Cox proportional hazards models were performed to identify significant prognostic predictors. The hazard ratio (HR) and 95% confidence interval (CI) were calculated. The significant variables with p <0.1 defined in univariate survival analyses (by the log rank test) were included for the multivariate analyses to validate the prognostic value of the P-LCR. A p-value less than 0.05 (2-tailed) indicated a statistical significance.

## Results

### Patient Characteristics

**Figure 1:**
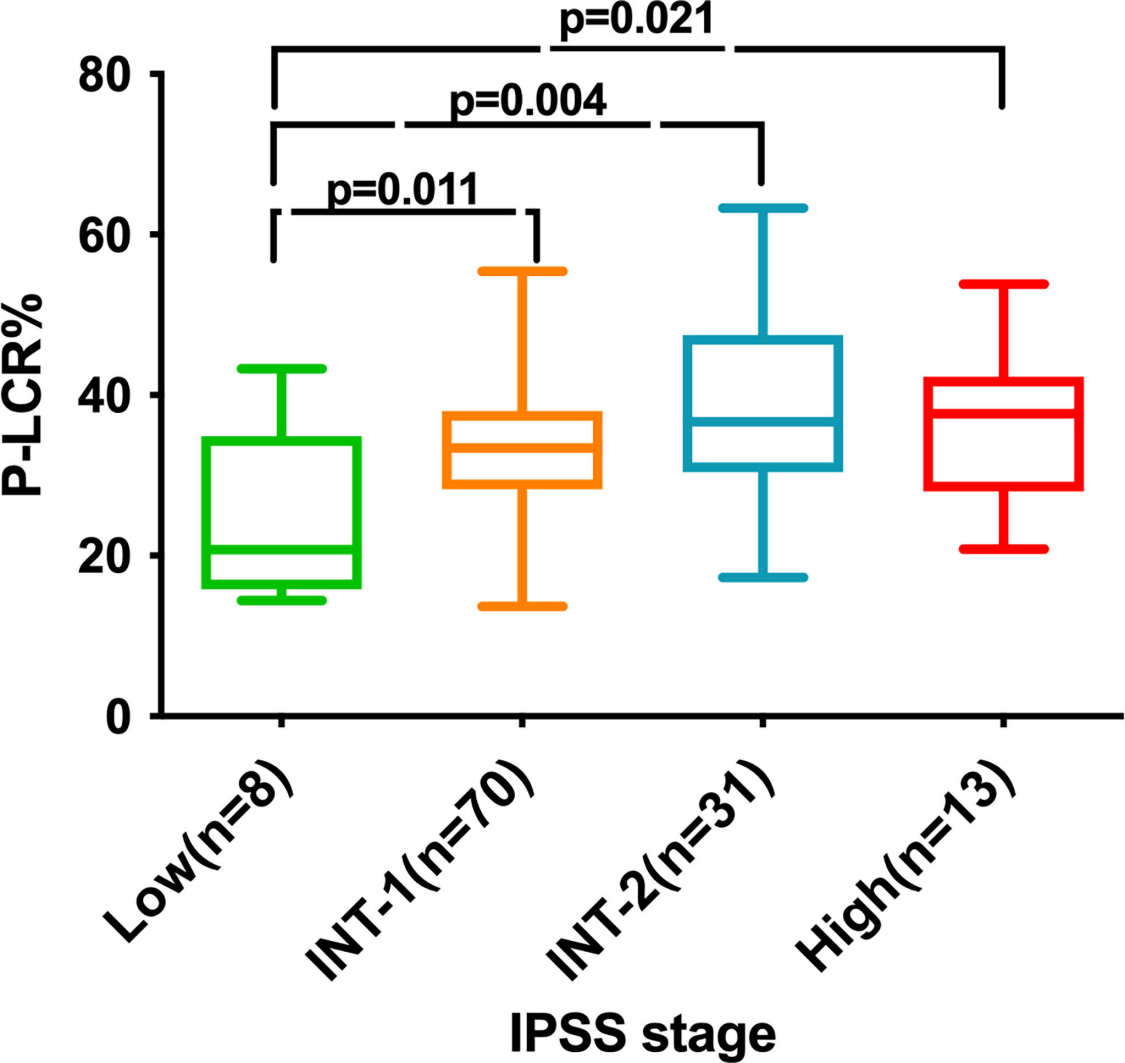
P-LCR level of 122 MDS patients at diagnosis according to the IPSS.

A total of 122 newly diagnosed MDS patients were included in our cohort. The median follow-up time was 48.27 months (range, 21.50–59.83 months). A total of 85 patients died. The demographics of patients are summarized in **Table 1**. The median age was 64.48 (26–88) years, and 84 (68.9%) were men. Subjects were classified as P-LCR^low^ (<36.7%; N = 74) or P-LCR^high^ (≥36.7%; N = 48) cohorts. The distribution of characteristics such as age, gender, WHO subtype, Hb level, bone marrow blast%, WBC count, ANC count, PLT count, IPSS subgroups, and treatment were not different between the two groups. Patients with IPSS low-risk disease presented with a lower P-LCR compared to INT-1 (p=0.011), INT-2 (p=0.004), and high risk (p=0.021) patients (**Figure 1**).

**Table 1:** Characteristics of 122 subjects with MDS.

Characteristics	Total (n=122)	P-LCR^low^ (n=74)	P-LCR^high^ (n=48)	P
Age, years, median (IR)	65 (59-72)	66 (60-73)	65 (55.25-71)	0.422
Men (%)	84 (68.9)	51 (68.9)	33 (68.8)	0.984
WHO subtype (%)				0.220
MDS-SLD	3 (2.4%)	1 (1.3)	2 (4.1)	
MDS-RS-SLD	11 (9%)	7 (9.4)	4 (8.3)	
MDS-MLD	19 (15.5)	8 (10.8)	11 (22.9)	
MDS-RS-MLD	5 (5)	1 (1.3)	4 (8.3)	
MDS-EB1	26 (21.3)	18 (24.3)	8 (16.6)	
MDS-EB2	35 (28.6)	23 (31.0)	12 (25)	
MDS with isolated 5q-	2 (1.6)	1 (1.3)	1 (2.0)	
MDS-U	21 (17.2)	15 (20.2)	6 (12.5)	
Hb, g/L, median (IR)	68 (56-83)	72 (56-87)	67.5 (56.25-77)	0.261
Bone marrow blast%, median (IR)	4 (0-8)	4 (0-6.5)	3.75 (0-8)	0.803
WBC, ×10^9^/L, median (IR)	2.46 (1.535-3.745)	2.17 (1.51-3.73)	2.47 (1.6-4.065)	0.487
ANC, ×10^9^/L, median (IR)	1.08 (0.57-1.955)	1.09 (0.45-1.99)	1.06 (0.695-1.74)	0.570
PLT, ×10^9^/L, median (IR)	45 (20.75-126.5)	38 (19-132)	49 (21.5-106.75)	0.777
IPSS risk group (%)				0.102
Low	8 (6.6)	7 (9.5)	1 (2.1)	
INT-1	70 (50.4)	46 (62.2)	24 (50.0)	
INT-2	31 (25.4)	15 (20.3)	16 (33.3)	
High	13 (10.7)	6 (8.1)	7 (14.6)	
Treatment (%)				0.291
Immunosuppressive drugs	5 (4.1)	4 (5.4)	1 (2.1)	
EPO ± G-CSF and/or RBC transfusions	67 (54.9)	37 (50.0)	30 (62.5)	
HMAs+CAG/HAG	21 (17.2)	16 (21.6)	5 (10.4)	
HMAs	27 (22.1)	15 (20.3)	12 (25)	
Allotransplant	2 (1.6)	2 (2.7)	0 (0)	

ANC, absolute neutrophil count; CAG/HAG, cytarabine combined with aclacinomycin or homoharringtonine and G-CSF; EPO, erythropoietin; G-CSF, granulocyte-colony-stimulating factor; Hb, hemoglobin; IPSS, International Prognostic Scoring System; IR (InterQuartile Range**);** MDS, myelodysplastic syndrome; MDS-EB1, MDS with excess blasts-1; MDS-EB2, MDS with excess blasts-2; MDS-MLD, MDS with multilineage dysplasia; MDS-SLD, MDS with single lineage dysplasia; MDS-RS-MLD, MDS with ring sideroblasts with multilineage dysplasia; MDS-RS-SLD, MDS with ring sideroblasts with single lineage dysplasia; MDS-U, MDS unclassifiable; PLT, platelet count; RBC, red blood cell; RDW, red blood cell distribution width; WHO, World Health Organization.

### Association Between P-LCR Level and Clinical Outcomes

**Figure 2:**
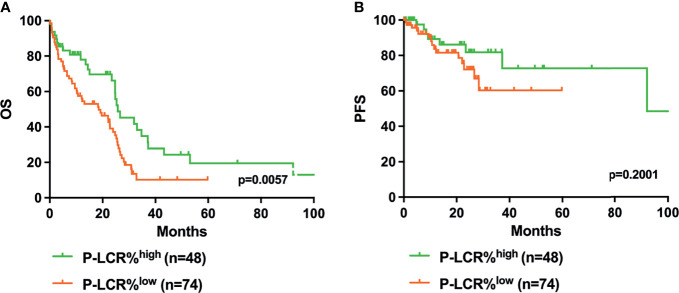
Higher P-LCR was associated with better OS **(A)** but not with better PFS **(B)**.

**Figure 3:**
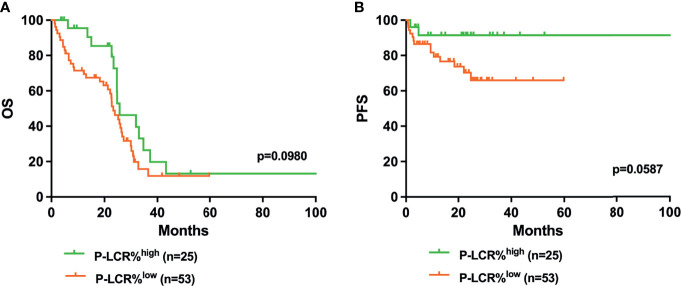
Higher P-LCR was not associated with better OS **(A)** and better PFS **(B)** in lower risk MDS groups (lower risk MDS group was defined as IPSS=low risk+INT-1).

**Figure 4:**
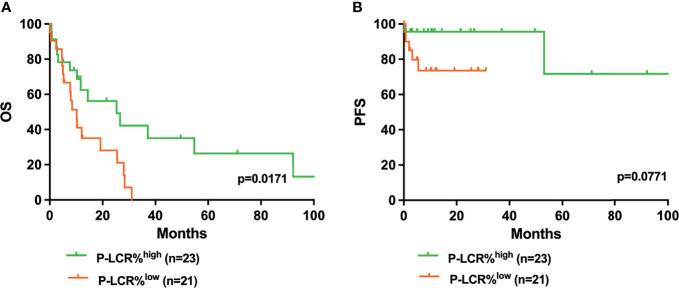
Higher P-LCR was associated with better OS **(A)** but not with better PFS **(B)** in higher risk MDS groups (higher risk MDS group was defined as IPSS=INT-2+high risk).

Kaplan–Meier survival curves were performed to compare the prognosis between P-LCR^low^ and P-LCR^high^ groups by the log rank test. The data showed that P-LCR^low^ was associated with worse OS compared to P-LCR^high^ patients (median OS, 18.53 months versus 25.77 months, p=0.0057, **Figure 2A**). A similar tendency was observed in PFS but with no statistical difference (p=0.2001, **Figure 2B**). Subgroup analyses were done in lower risk groups (including low risk and INT-1) and higher risk groups (including INT-2 and high risk). Patients with lower risk were expected to have better prognosis. The analyses of relationship between the P-LCR and prognosis in lower risk patients indicated that there was no statistical significance (p=0.0980 and p=0.0587; **Figure 3**). The association was also explored in higher risk groups. The results show that patients in higher risk groups with low P-LCR have shorter OS compared to those with high P-LCR, respectively (p=0.0171, **Figure 4**). There was no similar observation in PFS analysis (p=0.0771, **Figure 4**).

### Univariate Analyses for PFS and OS

Univariate analyses were performed to investigate the prognostic factors affecting disease progression and death (**Table 2**). The baseline P-LCR level (HR 0.578, 95%CI 0.363–0.919, p=0.020), bone marrow blast level (HR 1.083, 95%CI 1.042–1.126, p<0.001), platelet count (HR 0.997, 95%CI 0.994-0.999, p=0.018), and IPSS scores (HR 1.530, 95%CI 1.160-2.018, p=0.003) were potential risk factors for poor OS. The P-LCR was also prognostic for PFS (HR 0.263, 95%CI 0.087-0.794, p=0.018).

**Table 2:** Univariate analyses for PFS and OS in 122 MDS patients in relation to prognostic parameters.

	PFS			OS		
	HR	95%CI	P	HR	95%CI	P
P-LCR (<36.7 VS ≥36.7)	0.263	0.087-0.794	0.018	0.578	0.363-0.919	0.020
Sex	0.460	0.206-1.030	0.059	1.046	0.662-1.653	0.864
Age (year)	0.998	0.966-1.031	0.898	1.015	0.996-1.033	0.116
bone marrow blast (%)	1.001	0.914-1.096	0.986	1.083	1.042-1.126	P<0.001
WBC (×10^9^/L)	1.063	0.900-1.256	0.472	1.051	0.955-1.157	0.309
ANC (×10^9^/L)	1.072	0.856-1.342	0.547	1.023-	0.893-1.173-	0.742
Hb (g/L)	1.014	0.997-1.032	0.116	1.007	0.997-1.017	0.148
PLT (×10^9^/L)	0.993	0.987-1.000	0.057	0.997	0.994-0.999	0.018
IPSS scores	1.319	0.781-2.228	0.300	1.530	1.160-2.018	0.003

CI, confidence interval; HR, hazard ratio; PLT, platelet count; PFS, progression-free survival; OS, overall survival.

### Multivariate Analyses for PFS and OS

All variables with P < 0.1 in univariate analyses were included in multivariate analyses. The P-LCR, gender, bone marrow blast, PLT count and IPSS scores were the prognostic-related risk factors. We performed multivariate Cox regression analyses with the clinical variables based on the risk factors above. The results showed that the P-LCR at diagnosis in patients with MDS is an independent predictor for PFS in the total cohort in multivariate analyses (HR 0.212, 95%CI 0.064-0.701, p=0.011, **Table 3**) after the adjustment with gender, age, blasts, and PLT count. The P-LCR level (HR 0.464, 95%CI 0.284-0.757, p=0.002) was a potential prognostic factor for OS in multivariate analyses are shown in **Table 3**.

**Table 3:** Multivariate analyses for PFS and OS in 122 MDS patients in relation to prognostic parameters.

		PFS			OS	
	HR	95%CI	P	HR	95%CI	P
P-LCR	0.212	0.064-0.702	0.011	0.464	0.284-0.757	0.002
Sex	0.454	0.202-1.020	0.056	–	–	–
IPSS scores	–	–	–	1.304	0.863-1.972	0.208
Bone marrow blast (%)	–	–	–	1.049	0.992-1.110	0.094
PLT (×10^9^/L)	0.212	0.064-0.702	0.028	0.997	0.994-1.000	0.043

## Discussion

The present study showed that the P-LCR level at diagnosis was associated with the prognosis in patients with MDS. As far as we are aware, this study is the first report showing the P-LCR as an independent prognostic variable in MDS. We found that the patients whose P-LCR level was less than 36.7% at diagnosis experienced shorter overall survival compared to patients with a high P-LCR. There seems to be a similar tendency in the relationship between the P-LCR and PFS. PFS in the P-LCR^low^ group seemed to be shorter compared to the P-LCR^high^ group, although it was not statistically significant in the presented data. Subgroup analyses indicated that the P-LCR has a higher prognostic value in patients with higher risk.

Univariate and multivariate analyses were also performed to investigate the prognostic factors affecting disease progression and death. Results indicated that the P-LCR is a prognostic parameter in patients with MDS. Although the mechanism of action is still unclear, there is a consensus that the P-LCR is a potential biomarker, which can be quickly and accurately detected in peripheral blood test. Moreover, the baseline platelet count seemingly has a prognostic impact in MDS. While various studies have evaluated the prognostic significance of thrombocytosis in cancers, such as colon cancer, ovarian cancer, and hepatocellular cancer (13–15), no significance for platelet count in MDS has been reported. Gender seems to influence the prognosis of MDS patients; the same conclusion was also found in previous studies (16, 17). Some possible explanations for that might include 1) the increased comorbidities upon diagnosis in male patients, which limited preferred choices and the aggressiveness of treatment options (18, 19), while large cohort-based studies confirmed that MDS patients with the above comorbidities had significantly greater risk of death than those without comorbidities (20); 2) male MDS patients might be associated with molecular abnormalities such as faster methylome aging and shorter telomeres (21), both of which often correlate with shorter survival (22).

The present data suggested that the elevated P-LCR level at diagnosis was associated with better prognosis in MDS patients, especially for those with higher risk disease. We speculate that the reasons may be that, firstly, more large platelets are involved in the neoplastic consumed, thereby speeding up the progression of the tumor (23); secondly, the degree of platelet activation affects various effector factors, such as Vascular Endothelial Growth Factor (VEGF), Epidermal Growth Factor (EGF), transforming growth factor-beta (TGFβ), Platelet-derived growth factor (PDGF), and Interleukin-6 (IL-6), that impact vascular maturation in the tumor microenvironment and mediate the invasion of cancer cells (24), which are associated with survival in MDS. Third, large platelet cells might be associated with greater platelet–tumor complex formation; therefore, patients with a high P-LCR could gain more benefit from antiplatelet drugs than could patients with low P-LCR levels (25).

Platelets play a critical role in the development and progression of different cancers by promoting cancer cell proliferation, survival, angiogenesis, and metastasis (26, 27). There have been several studies revealing that platelet indices are associated with prognosis in patients with various diseases. Thrombocytosis was related to poor prognosis in several cancers, such as ovarian, gastric, lung, breast, hepatocellular, and bladder cancer (28–33). Higher platelet distribution width (PDW) was correlated with unfavorable prognosis in ovarian, and breast cancer (34, 35). Moreover, the mean platelet volume (MPV) level was evaluated for association with the development of diabetes mellitus (36) and outcomes of cancer patients (37, 38). However, the impact of the P-LCR in MDS is unclear.

The platelet–large cell ratio (P-LCR) is an indicator of circulating larger platelets (>12 fl) and the best tool to assess megakaryocyte activity (24). A large platelet is somehow a representative marker of immature platelets; thus, the lower P-LCR observed in MDS patients in comparison to the respectively high group suggests increased platelet maturity. These findings are also similar to those of Renate Asare et al. (39), whose results suggested that there are significantly (p<0.05) lower levels of P-LCR in children with Burkitt lymphoma than the controls. The mechanisms for the phenomenon remain uncertain. Psaila et al. (40) found that patients with AML/MDS had smaller platelets and lower *in vivo* platelet activation and *ex vivo* platelet reactivity than patients with immune thrombocytopenia. All these studies prompt that the flow cytometric analyses of platelet function and cell parameter analyzers could establish the expression of the platelet indices and reflect the megakaryocyte activity, so as to judge the marrow environment indirectly. The P-LCR has good prognostic evaluation efficiency and could also reflect the changes of the general states of MDS from different aspects. As an easily obtained index, it may help to more accurately evaluate the prognosis of MDS.

## Conclusions

This is the first documentation on the prognostic value of P-LCR in patients with MDS with long-term follow-up. However, our data are preliminary; further prospective analyses and the mechanism studies are necessary.

## Data Availability Statement

The original contributions presented in the study are included in the article/**Supplementary Material**. Further inquiries can be directed to the corresponding authors.

## Ethics Statement

The studies involving human participants were reviewed and approved by Institutional Review Committee of Huai’an No.1 People’s Hospital. Written informed consent for participation was not required for this study in accordance with the national legislation and the institutional requirements.

## Author Contributions

LY designed the study. QC and YC wrote the manuscript. YZ collected data. LZ and KC were responsible for the tables. ZH was responsible for the figures. CW and LY modified the manuscript.

## Funding

This work was funded by Science and Technology Fund of Huaian City [grant # HAB202020] and Commission of Health of Jiangsu Province [grant # 2019082].

## Conflict of Interest

The authors declare that the research was conducted in the absence of any commercial or financial relationships that could be construed as a potential conflict of interest.

## Publisher’s Note

All claims expressed in this article are solely those of the authors and do not necessarily represent those of their affiliated organizations, or those of the publisher, the editors and the reviewers. Any product that may be evaluated in this article, or claim that may be made by its manufacturer, is not guaranteed or endorsed by the publisher.
