# Supplementary material for: Prognostic Impact of Platelet-Large Cell Ratio In Myelodysplastic Syndromes
Source: Front Oncol. 2022 Apr 1;12:846044. doi: 10.3389/fonc.2022.846044 (PMC9010610; doi:10.3389/fonc.2022.846044)

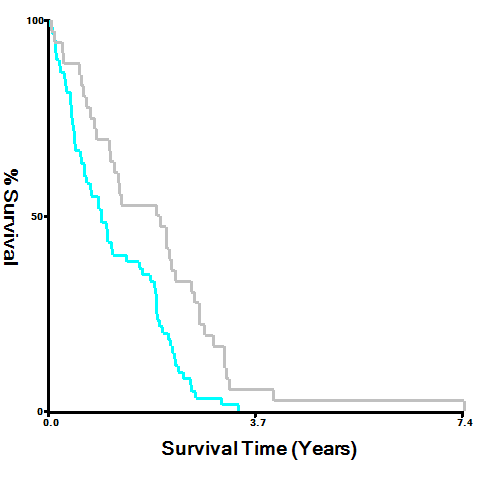

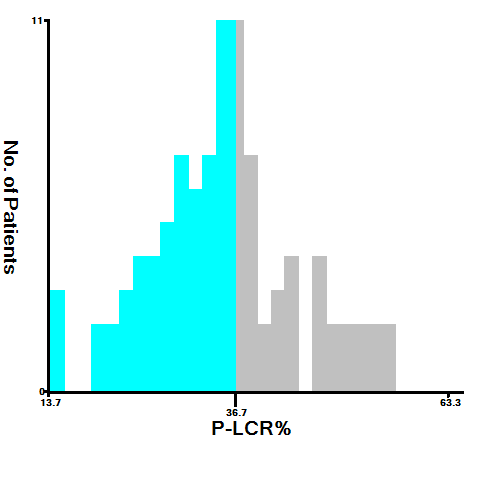


Figure S1 X-tile analysis of prognosis based on P-LCR.

X-tile analyses of overall survival (OS) were performed using patients’ data to determine the optimal cut-off values for P-LCR. Patients' information of OS was input to X-tile software with the number of P-LCR, and the optimal cutoff points for the P-LCR were analyzed by the computer program. The optimal cut-off values highlighted by the black circles in left panels are shown in histograms of the entire cohort (middle panels), and Kaplan–Meier plots are displayed in right panels. The optimal cut-off value for P-LCR was 36.7, which divided the patients into two groups: “P-LCR ＜36.7” group and “P-LCR ≥36.7” group.


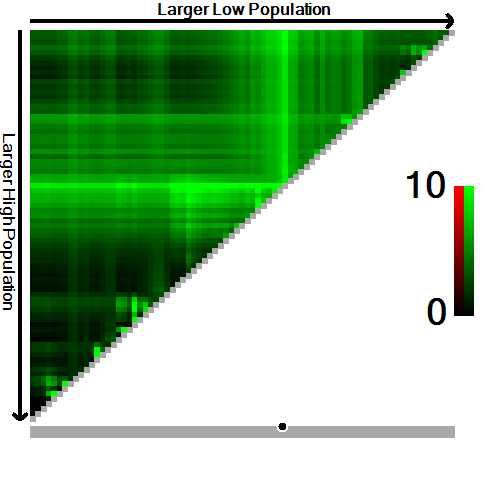

Supplement: Supplementary file 1 [file DataSheet_1.docx]
